# Very long-chain fatty acids accumulate in breast cancer tissue and serum

**DOI:** 10.1186/s12935-025-03928-2

**Published:** 2025-08-04

**Authors:** Alicja Pakiet, Michalina Ciosek, Oliwia Lange, Katarzyna Duzowska, Agata Janczy, Małgorzata Kapusta, Yelyzaveta Razghonova, Marcin Ekman, Anna Abacajew-Chmyłko, Paweł Kabata, Adriana Mika

**Affiliations:** 1 Department of Environmental Analysis, Faculty of Chemistry, University of Gdansk, Wita Stwosza 63, Gdansk, 80-308 Poland; 2 Department of Surgical Oncology, Transplant Surgery and General Surgery, Faculty of Medicine, Medical University of Gdansk, Mariana Smoluchowskiego 17, Gdansk, 80-214 Poland; 3 Department of Pharmaceutical Biochemistry, Faculty of Pharmacy, Medical University of Gdansk, Debinki 1, Gdansk, 80-211 Poland; 4 Department of Food Commodity Science, Faculty of Health Sciences with the Institute of Maritime and Tropical Medicine, Medical University of Gdansk, Debinki 7, Gdansk, 80-211 Poland; 5 Bioimaging Laboratory, University of Gdansk, Wita Stwosza 59, Gdansk, 80-308 Poland; 6 Department of Gynecology, Obstetrics and Neonatology, Faculty of Medicine, Medical University of Gdansk, Mariana Smoluchowskiego 17, Gdansk, 80- 214 Poland; 7 Department of General Surgery, Copernicus Mamma Centrum Copernicus LLC, Al. Zwycięstwa 31/32, Gdańsk, 80-210 Poland

**Keywords:** Breast cancer, Fatty acids, Very-long chain fatty acids, VLCFAs, Fatty acid elongase 1, ELOVL1

## Abstract

**Background:**

Breast cancer (BC) remains one of the most common cancers with relatively high mortality and is associated with alterations in fatty acid (FA) metabolism. While typical FAs have been extensively studied, there is increasing evidence for a potential role of very long chain fatty acids (VLCFAs) in cancer growth and progression.

**Methods:**

This study involved 54 BC patients from whom samples of malignant tumor, normal fibroglandular tissue, and breast adipose tissue were collected. Their FA content was analyzed by gas chromatography-mass spectrometry. The expression of fatty acid elongases (ELOVLs) and FA-transporting proteins was analyzed in the tissues by RT-PCR and immunofluorescence. Results: Higher levels of saturated and monounsaturated VLCFAs were found in BC tissues compared to normal tissues (*p* < 0.001) and in patients’ blood compared to healthy controls blood (*p* < 0.001). However, the level of VLCFAs was lower in BC adipose tissue compared to healthy control adipose tissue (*p* < 0.001). Interestingly, there were no obvious differences in ELOVL1 mRNA or protein levels between normal and cancer tissues. Conclusions: Elevated levels of VLCFAs in BC tissue are the result of lipid uptake from outside the tumor rather than in situ synthesis.

**Supplementary Information:**

The online version contains supplementary material available at 10.1186/s12935-025-03928-2.

## Introduction

Female breast cancer (BC) remains one of the most common diseases worldwide and is one of the most frequent causes of death in female cancer patients [1, 2].

Although much of the research has focused on the role of conventional fatty acids (FAs) in BC, a growing number of studies suggest that very long-chain fatty acids (VLCFAs) may play a critical role in tumor growth and spread [3–6]. VLCFAs are usually defined as FAs with a carbon chain length of 20 or more and can be both ingested from food and synthesized in vivo [7]. The endogenous elongation of FAs to VLCFAs occurs in the endoplasmic reticulum, where the condensation step is catalyzed by one of the seven elongases (ELOVL1-7), which have different substrate preferences. The elongation of saturated and monounsaturated FAs is facilitated by ELOVL1, 3, 6 and 7, ELOVL2 and 5 are involved in the elongation of polyunsaturated FAs (PUFAs), while ELOVL4 acts upon various FAs with over 22 carbon chain lengths. The isoform mainly responsible for the synthesis of saturated and monounsaturated VLCFAs is ELOVL1 [8]. The unusually long acyl chains confer different properties and functions to VLCFAs compared to their shorter counterparts. For example, C24 FA-containing sphingolipids affect the physical properties of cell membranes [7]. VLCFAs have been shown to induce endoplasmic reticulum stress [9]. Moreover, recent research on pro-tumorigenic activities of peroxisomes underlines the link between the metabolism of VLCFAs and cancer, as their β-oxidation occurs almost exclusively in peroxisomes [10].

The detailed study of the specific FA composition [6, 11–15] of tissues and biofluids from cancer patients, especially with the advent of mass-spectrometry-based analysis, is an avenue that can provide useful insights into lipidomic disturbances in patients’ bodies. This in turn can be utilized to identify specific cancer-associated biomarkers and uncover disrupted pathways of lipid metabolism that can be addressed with therapy.

The aim of the study was to analyze the FA profiles in serum and breast tissue of BC patients, focusing on the levels of VLCFAs, that could have potential implications for the development of new diagnostic and therapeutic strategies. We also tried to explain the observed changes at the molecular level by examining the expression of the ELOVLs genes in normal and neoplastic tissues from BC patients.

## Materials and methods

### Subjects of the study

The study included 54 female patients with BC, confirmed by screening mammography or ultrasound, treated at the Department of Surgical Oncology, Transplant Surgery and General Surgery of the Medical University of Gdansk. Patients with a presence of only ductal carcinoma in situ (DCIS) were excluded. The control group consisted of 34 women without BC, who presented to the study department with benign breast disease. The tissue samples taken from BC patients at the time of surgery were malignant tumor tissue (cancer tissue, *n* = 54), normal fibroglandular breast tissue removed from the tumor margin (normal tissue, *n* = 54) and peripheral adipose tissue (*n* = 34). Representative images of hematoxylin and eosin (H&E) staining of cancer and normal mammary gland tissue are shown in Supplementary Figure S1. Adipose tissue was also collected from 10 control subjects (control adipose tissue) at the time of non-malignant tumor removal or breast reduction surgery. The tissue samples were immediately frozen in liquid nitrogen and stored at −80 °C until the analysis. 5 ml of blood was drawn from all study participants after an overnight fast, and serum was obtained by centrifugation. All serum samples (53 sera from BC patients and 34 control sera) were stored at −80 °C. Directly before analyses all samples were weighed and immediately placed in appropriate buffers or solvent mixtures. During this step, care was taken to ensure that the samples were not thawed. The routine laboratory blood parameters were determined in the Central Clinical Laboratory of the Medical University of Gdansk.

### GC-MS analysis of the fatty acids

Approximately 50 mg of the tissue was homogenized in an extraction mixture according to Folch et al. [16], i.e. chloroform-methanol (2:1, v/v). The same mixture was used for extracting lipids from 300 µl aliquots of serum. The extracted total lipids were then dried under N_2_ stream, reconstituted in 1 ml of 0.5 M KOH, and hydrolyzed at 90° C. After 3 h the mixtures were acidified with 0.5 ml 6 M HCl. 1 ml H_2_O was added, and the free FAs were extracted with three portions of 1 ml *n*-hexane, after which the organic phase was evaporated under N_2_ stream. The free fatty acids were then derivatized to methyl esters with 0.5 ml of 10% BF_3_ solution in methanol and incubation at 55 °C for 1.5 h. The FA esters were then extracted from the derivatization mixture by adding 1 ml of H_2_O and three portions of 1 ml of *n*-hexane. The organic phase was then dried under N_2_ stream and stored at −80 °C until analysis.

The FA methyl esters were reconstituted in dichloromethane and analyzed with a GC-EI-MS QP 2010SE (Shimadzu, Kyoto, Japan). Chromatographic separation was performed on a Zebron ZB-5MSi capillary column (30 m x 0.25 i.d. x 0.25 μm film thickness). The injector temperature was set to 300 °C and helium with a column head pressure of 100 kPa was used as the carrier gas. The column oven temperature was ramped from 125 °C to 285 °C at 4 °C per min. The total run time of the analysis was 40 min. The mass spectrometer operated in full scan mode, the mass scan range was m/z 45–700 and electron impact source was set to 70 eV. The identification of FAs was aided by using the reference standard mixture 37 FAME Mix (Sigma-Aldrich, St. Louis, MO, USA) and the NIST/EPA/NIH Mass Spectral Library (NIST 11) − 2012 Mass Spectral Database for Windows, Standard Reference Data Program (U.S. Department of Commerce. National Institute of Standards and Technology, Gaithersburg, MD, USA). The 19-methylarachidic acid was added as an internal standard. FA levels are expressed as % of all FA identified by MS. It was calculated from the signal intensity.

### Evaluation of oxidative damage and non-esterified fatty acids

Lipid peroxidation was determined using Thiobarbituric Acid Reactive Substances (TBARS) Assay Kit from Cayman Chemical Company (Ann Arbor, MI, USA, cat. no. 10009055). Carbonyl content was measured using the Protein Carbonylation Content Assay Kit (cat. no. MAK094) and non-esterified fatty acid (NEFA) content was measured using the Free Fatty Acid Quantitation Kit (cat. no. MAK044), both purchased from Sigma-Aldrich (St. Louis, MO, USA). Assays were performed according to the manufacturers’ instructions, each sample was run in duplicate, and the absorbance of the samples and calibration curves were measured using a Synergy HT multiplate microplate reader (BioTek, Winooski, VT, USA). These analyses were performed in the tissues of BC patients (cancer tissue and normal tissue, see Supplementary Figure S2) and in the serum of BC patients and the control group (see Table 1).

### Evaluation of mRNA levels by real-time PCR

Frozen tissue samples were used for extraction of total RNA using the RNeasy Plus Universal MiniKit (Qiagen, Hilden, Germany, 73404) according to the manufacturer’s guidelines. The concentration and purity of extracted RNA was checked using a NanoDrop spectrophotometer (Thermo Fisher Scientific, Waltham, MA, USA) and an Experion™ automated gel electrophoresis system (Bio-Rad Laboratories, Hercules, CA, USA). Only RNA pairs isolated from normal and cancer tissue that had a RQI above 7 out of 10 in the Experion RNA integrity analysis were used for the analysis. The RNA was then reverse transcribed into complementary DNA (cDNA) using the RevertAid First Strand cDNA Synthesis Kit (Thermo Fisher Scientific, Waltham, MA, USA, K1622) and stored at −20 °C until further processing. Quantitative real-time PCR analysis was performed using the CFX Connect Real-Time PCR Detection System (Bio-Rad) in combination with the SensiFAST SYBR No-ROX Kit (Meridian Bioscience, Cincinnati, OH, USA, BIO-98020). Cyclophilin A was selected as the reference gene. The levels of cyclophilin A did not differ between cancer tissue and normal breast tissue from BC patients (mean Ct values 23.8 ± 1.1 vs. 23.2 ± 0.8, not significant). The primers targeting ELOVLs: 1, 2, 3, 4, 5, 6, 7 and the CD36 and FATP2 genes were synthesized by Genomed S.A. (Warsaw, Poland), and their sequences are shown in Supplementary Table 1. The data were analysed using the 2^−△△Ct^ relative quantification method.

### Evaluation of ELOVL1 protein level by Immunofluorescence method

Tissue pairs from the same patients (tumors and normal breast tissue) were fixed in 4% paraformaldehyde in 0.2 M PBS buffer (pH 7.4), dehydrated through a graded series of ethanol (10–100%), and embedded in low melting point (37℃) polyester wax (Steedman’s wax, i.e., a 9:1 (w/w) mixture of polyethylene glycol 400 distearate and cetyl alcohol, Sigma-Aldrich), method modified from Merchant et al. [17]. Samples were cut into 6–7 μm thick sections using MICROM HM 350 microtome, dewaxed in ethanol and stained with standard H&E staining tissues protocol (Mayer’s hematoxylin and Eosin Y). They were then cleared in xylene and mounted in DPX. Samples were visualized using Leica DM 6000B epifluorescence microscope with motorized z-axis in brightfield illumination mode. For the ICC-IF protocol, sections were permeabilized with 0.1% Triton X-100 in PBS buffer (Sigma Aldrich) for 10 min at RT and blocked in 4% BSA in PBS (Sigma Aldrich) for 1 h at RT. Elovl1 was detected using a rabbit primary antibody against ELOVL fatty acid elongase 1 (Sigma Aldrich, Prestige antibodies HPA056557) overnight at 4˚C at a dilution of 1:50, followed with secondary antibody conjugated to DyLight 555 (1:800). Negative controls were prepared by omitting the primary antibody step (Supplementary Figure S3). Samples were counterstained with 7 µg/ml DAPI and cover-slipped with MOWIOL mounting medium. Tissues were visualized using the Leica Stellaris 5 WLL confocal microscope. Mean fluorescence intensity of glandular and tumor cells was recorded from freely selected ROIs and measured with the LAS X software at the same parameters as the recorded photographs (63x magnification, speed = 200 Hz, 13.99% laser intensity and gain = 31.3).

### Data handling

VLCFAs are defined as follows: even-chain VLCFAs ≥ 20:0; odd-chain VLCFAs ≥ 21:0; saturated VLCFAs are a sum of even- and odd-chain VLCFAs; monounsaturated VLCFAs ≥ 20:1. Very long chain PUFAs were not included in VLCFA group, since they have different biological properties than very long chain SFAs and MUFAs, and in fact most of PUFAs have ≥ 20:0 carbons in their chain. Values are expressed as mean ± SD. Comparisons between groups were performed after removal of the outliers identified using the 1.5 x IQR (interquartile range) rule. For paired tissue samples (cancer tissue and normal breast tissue from BC patients), the paired Student’s t-test was used if the data were normally distributed (confirmed with the Shapiro-Wilk test) and the variance was equal (Fisher test), and for data that did not met this conditions, the Wilcoxon Signed Ranks test was used. Comparisons between BC patients and control subjects were performed with the t-test for equal or unequal variance (checked with the Fisher test) and for the data without normal distribution the Wilcoxon Rank Sum test was used. To account for the potentially confounding effect of body mass index (BMI), each serum FA was analysed using analysis of covariance (ANCOVA), with group as the main factor and BMI as a continuous covariate. To control for the false discovery rate due to multiple testing across fatty acids, p-values for the group effect were adjusted using the Benjamini–Hochberg procedure. For multiple comparisons ANCOVA was followed by post hoc pairwise comparisons between subtypes using estimated marginal means (EMMs), adjusted using Tukey’s method to correct for multiple testing. To evaluate the discriminatory power of selected FA groups receiver operating characteristic (ROC) analyses were performed, with adjustment for BMI as a potential confounder. To mitigate overfitting and improve estimate robustness, 5-fold cross-validation was applied. Predicted probabilities from each fold were combined to generate ROC curves, with the area under the curve (AUC) and 95% confidence intervals computed using DeLong’s method. The results were considered significant at *p* < 0.05. Statistical analysis was performed in R version 4.2.0 using the R packages stats, car, emmeans, dplyr, pROC, ggplot2, tidyr, and ggpubr. The principal least squares discriminant analysis (PLS-DA) models were generated using the MetaboAnalyst 4.0 software (https://www.metaboanalyst.ca/). Prior to these analyses the data were logarithmically transformed and scaled. The mean was centered and divided by the standard deviation of each variable.

## Results

###  Anthropometric, clinical and biochemical characteristics of study participants

The anthropometric, clinical and biochemical data of the BC patients and control subjects are shown in Table 1. The BC group exhibited slightly elevated glucose, markedly higher insulin and HOMA-IR and C-peptide, and elevated NEFA when compared with control group. The triglyceride (TG) levels did not differ significantly between the groups (Table 1).

**Table 1:** Characteristics of study patients

	Breast cancer patients	Healthy control subjects	*p*-value
N	54	34	N/A
BMI	28.6 ± 14.4	25.9 ± 13.4	0.043*
Age	59 ± 30	59 ± 23	0.997
Stage [n]			
I	21	N/A	N/A
II	25	N/A	N/A
III	3	N/A	N/A
Unknown	5	N/A	N/A
Histopathological type [n]			
Luminal A (ER + PR + HER2-)	20	N/A	N/A
Luminal B (ER + PR- HER2-)	22	N/A	N/A
Luminal B HER2+ (ER + PR- HER2+)	5	N/A	N/A
Non-luminal HER2+	1	N/A	N/A
Triple negative breast cancer	0	N/A	N/A
Other	6	N/A	N/A
Leukocytes [x10^9^/l]	6.71 ± 3.67	6.62 ± 2.96	0.914
Albumin [g/l]	39.2 ± 19.5	38.0 ± 16.4	0.361
Total protein [g/l]	68.8 ± 34.2	70.1 ± 30.4	0.614
HOMA-IR	6.86 ± 1.86	1.74 ± 0.26	0.009*
Glucose [mg/dl]	105 ± 63	89.4 ± 38.6	0.132
CRP [mg/l]	4.65 ± 6.80	2.73 ± 3.51	0.166
HDL-cholesterol [mg/dl]	52.7 ± 27.7	59.9 ± 32.0	0.055
LDL-cholesterol [mg/dl]	128 ± 70	120 ± 70	0.489
Cholesterol [mg/dl]	199 ± 102	198 ± 109	0.505
TG [mg/dl]	135 ± 102	124 ± 107	0.341
TBARS [µM]	0.16 ± 0.08	0.07 ± 0.07	< 0.001*
Carbonyl content [nmol carbonyl/mg protein]	0.61 ± 0.12	0.67 ± 0.10	0.040*
NEFA [mmol/l]	0.85 ± 0.39	0.31 ± 0.15	< 0.001*
Insulin [mU/ml]	22.4 ± 33.3	7.92 ± 5.24	0.028*
C-peptide [ng/ml]	4.80 ± 4.44	3.08 ± 1.50	0.574

Values are mean ± SD if not specified otherwise. * denotes significant p-value. CRP, C-reactive protein; ER+, estrogen receptor positive; HDL-cholesterol, high density cholesterol; HER2+, positive for human epidermal growth factor receptor 2; HOMA-IR, homeostatic model assessment for insulin resistance; LDL-cholesterol, low-density cholesterol; NEFA, non-esterified fatty acids; PR+, progesterone receptor positive; TBARS, thiobarbituric acid reactive substances; TG, triglycerides

### Accumulation of VLCFAs in breast cancer tissue

**Fig. 1:**
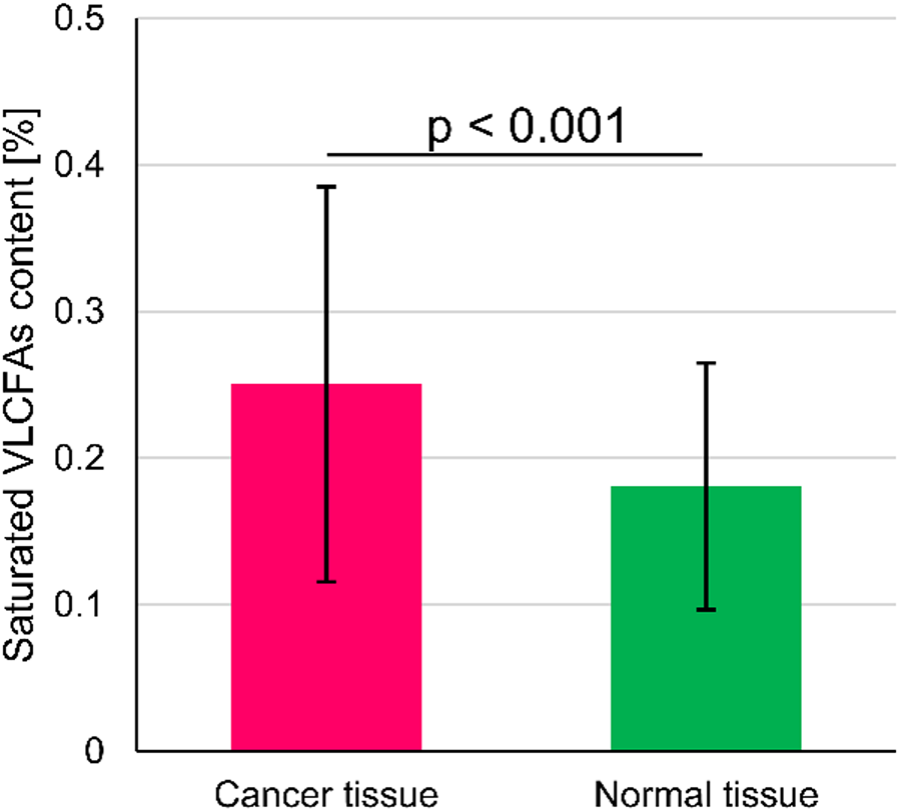
The levels of saturated VLCFAs in cancer tissue and normal mammary gland tissue from breast cancer patients

Mass spectrometry analysis of FA profiles in tissues from BC patients revealed a number of differences between cancer tissue and normal fibroglandular tissue (Table 2). Branched-chain FAs (BCFAs) content was increased in cancer tissues, mainly due to higher *anteiso*-BCFAs levels compared to the paired normal tissues. Among the n-6 PUFAs almost all FA were significantly more abundant in cancer tissues, except the linoleic acid (LA, 18:2 n-6). Cancer tissues also had increased levels of most abundant PUFAs n-3, namely α-linolenic acid (ALA, 18:3 n-3), docosahexaenoic acid (DHA, 22:6 n-3) and docosapentaenoic acid (n-3 DPA, 22:5 n-3). However, the most striking feature of the BC tissues was the presence of significantly higher amounts of saturated VLCFAs (Fig. 1). Similarly, most of monounsaturated VLCFAs were more abundant in BC tissues (Table 2). The levels of lignoceric (24:0) and nervonic acid (24:1) was increased approximately 2-fold. Additionally, we detected trace amounts of cerotic (26:0), pentacosanoic (25:0) and ximenic (26:1) acids in BC tissue, which were not present in normal breast tissues (Table 2).

There was no apparent relation between cancer progression or histopathologic type and VLCFAs, when analyses were stratified by cancer stage (Supplementary Table S2) or histopathologic type (Supplementary Table S3). We have only found significantly higher 25:0 in cancer tissue of BC patients with luminal B than those with luminal A histopathological type (Supplementary Table S3).

**Table 2:** Fatty acid content [%] in cancer tissue and normal mammary gland tissue from breast cancer patients

	Cancer tissue (*n* = 54)	Normal tissue (*n* = 54)	*p*-value
Even-chain VLCFAs	0.226 ± 0.016	0.165 ± 0.009	< 0.001*
20:0	0.141 ± 0.07	0.118 ± 0.060	0.004*
22:0	0.040 ± 0.037	0.024 ± 0.020	< 0.001*
24:0	0.039 ± 0.031	0.020 ± 0.017	< 0.001*
26:0	0.007 ± 0.009	traces	< 0.001*
ELOVL1 index	0.041 ± 0.002	0.032 ± 0.001	< 0.001*
Other ECFA	31.1 ± 0.406	30.9 ± 0.410	0.914
Sum of ECFA	31.3 ± 11.0	31.1 ± 10.9	0.359
Odd-chain VLCFAs	0.024 ± 0.003	0.016 ± 0.002	< 0.001*
21:0	0.010 ± 0.007	0.009 ± 0.008	0.436
23:0	0.013 ± 0.012	0.008 ± 0.008	< 0.001*
25:0	0.003 ± 0.005	traces	0.012*
Other OCFA	0.561 ± 0.018	0.556 ± 0.018	0.407
Sum of OCFA	0.585 ± 0.238	0.572 ± 0.233	0.179
Sum of *iso* BCFA	0.194 ± 0.082	0.195 ± 0.085	0.848
*anteiso* 17:0	0.140 ± 0.062	0.133 ± 0.059	0.039*
*anteiso* 19:0	0.075 ± 0.034	0.067 ± 0.027	0.005*
Other *anteiso* BCFA	0.053 ± 0.025	0.055 ± 0.027	0.153
Sum of *anteiso* BCFA	0.269 ± 0.114	0.254 ± 0.108	0.006*
4,8,12–14:0	0.012 ± 0.011	0.006 ± 0.006	< 0.001*
Sum of BCFA	0.473 ± 0.198	0.454 ± 0.194	0.020*
Sum of SFA	32.4 ± 11.3	32.1 ± 11.2	0.309
18:1	49.4 ± 16.9	50.2 ± 17.2	0.012*
Monounsaturated VLCFAs	1.02 ± 0.038	0.934 ± 0.030	0.054
20:1	0.934 ± 0.391	0.885 ± 0.359	0.178
22:1	0.043 ± 0.028	0.031 ± 0.016	0.001*
24:1	0.037 ± 0.057	0.019 ± 0.015	0.015*
26:1	traces	ND	-
Other MUFA	4.97 ± 0.191	4.09 ± 0.186	0.541
Sum of MUFA	55.4 ± 19.0	56.1 ± 19.2	0.020*
LA 18:2 n-6	9.94 ± 3.77	10.27 ± 3.82	0.018*
ARA 20:4 n-6	0.756 ± 0.716	0.455 ± 0.230	< 0.001*
DGLA 20:3 n-6	0.359 ± 0.301	0.226 ± 0.109	< 0.001*
EDA 20:2 n-6	0.243 ± 0.118	0.210 ± 0.095	0.001*
n-6 DPA 22:5 n-6	0.027 ± 0.023	0.020 ± 0.011	0.014*
AdA 22:4 n-6	0.273 ± 0.202	0.188 ± 0.103	< 0.001*
Other PUFA n-6	0.011 ± 0.006	0.011 ± 0.006	0.953
Sum of PUFA n-6	11.6 ± 4.33	11.4 ± 4.19	0.172
EPA 20:5 n-3	0.085 ± 0.094	0.059 ± 0.033	0.003*
DHA 22:6 n-3	0.195 ± 0.128	0.152 ± 0.094	< 0.001*
n-3 DPA 22:5 n-3	0.253 ± 0.151	0.202 ± 0.118	< 0.001*
Other PUFA n-3	0.033 ± 0.002	0.030 ± 0.002	0.135
Sum of PUFA n-3	0.567 ± 0.348	0.443 ± 0.244	< 0.001*
Sum of PUFA	12.2 ± 4.55	11.8 ± 4.35	0.069

Values are mean ± SD; ND, not detected; traces indicated that diagnostic ions were present, but the signal was too small to be integrated.Values are mean ± SD; ND, not detected; traces indicated that diagnostic ions were present, but the signal was too small to be integrated. $\:\:\mathbf{ELOVL}1\:\varvec{index}=\:\frac{20:0+22:0+24:0+26:0}{18:0}$, * denotes significant p-value. FA abbreviations: AdA, adrenic acid; ARA, arachidonic acid; BCFA, branched-chain fatty acid; DGLA, dihomo-γ-linolenic acid; DHA, docosahexaenoic acid; DPA, docosapentaenoic acid; ECFA, even-chain fatty acid; EDA, eicosadienoic acid; EPA, eicosapentaenoic acid; LA, linoleic acid; MUFA, monounsaturated acid; OCFA, odd-chain fatty acid; PUFA, polyunsaturated fatty acid; SFA, saturated fatty acid, VLCFA, very long chain fatty acid

Other ECFA = 10:0 + 12:0 + 14:0 + 16:0 + 18:0

Other OCFA = 11:0 + 13:0 + 15:0 + 17: 0 + 19:0

Other OCFA = 11:0 + 13:0 + 15:0 + 17: 0 + 19:0

Other anteiso BCFA = anteiso 15:0

Other MUFA = 10:1 + 12:1 + 14:1 + 16:1 + 17:1 + 19:1

Other PUFA n-6 = 16:2 n-6

Other PUFA n-3 = 18:3 n-3 + 20:4 n-3

**Fig. 2:**
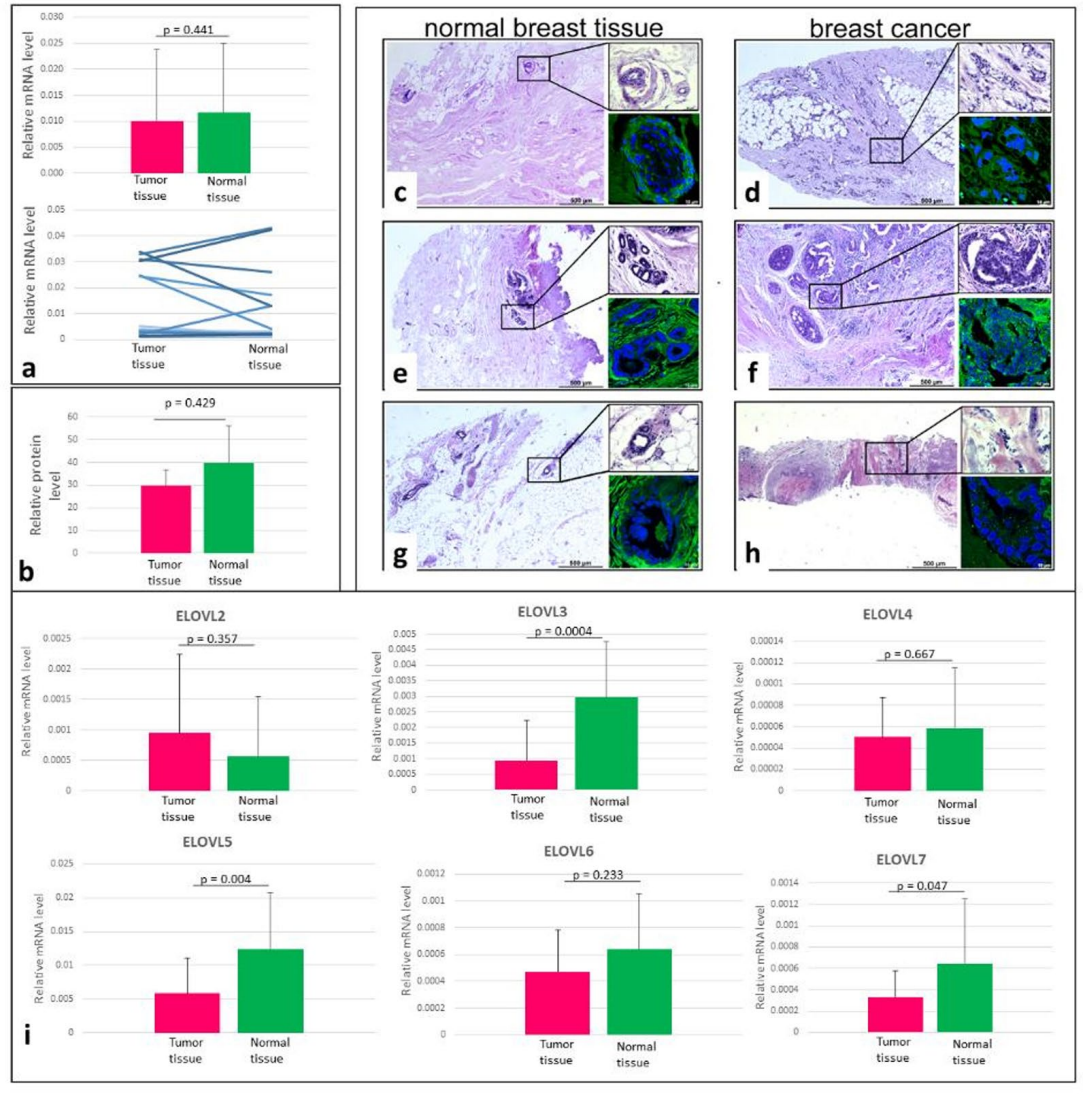
The expression of ELOVL1 and other ELOVLs in cancer tissue and normal mammary gland tissue from breast cancer patients. Relative mRNA levels (**a**), upper panel presents mean ± SD, the bottom panel represent results for each patient separately. Relative protein levels (**b**). Photographs after hematoxylin-eosin staining and immunofluorescence detection of elovl1 in representative mammary duct cells of normal breast tissue (**c**, **e**, **g**) and representative tumor cells (**d** and **h**-lobular carcinoma; **f** - duct carcinoma). Magnification 5x and higher magnification (20x). Relative mRNA levels of other ELOVLs (**i**)

One possible explanation for the high content of even-chain, saturated VLFCA in BC tissue is the higher expression of the rate-limiting enzyme of fatty acid elongation, elongase 1 (ELOVL1). To evaluate the expression of ELOVL1 in the patients’ tissues, mRNA levels were analysed by real-time PCR and protein levels by immunofluorescence. However, the results of these analyses show no obvious differences in ELOVL1 mRNA levels (Fig. 2A and B) or protein levels (Fig. 2C-H) between normal and cancer tissues.

We found that the ELOVL1 index was significantly higher in cancer tissue than in normal tissue (Table 2). However, it should be noted that when considering the ELOVL1 index as an indicator of ELOVL1 activity, it is assumed that all VLCFAs are produced in situ and no transport from outside the cell is taken into account. Therefore, this result should be treated with caution. We also measured the mRNA levels of other ELOVLs to see if other members of this family might be involved in elevated VLCFA levels. However, their mRNA levels were similar or even lower in cancer tissue than in normal mammary gland tissue (Fig. 2i). In turn, the levels of fatty acid transporting proteins FATP2 and CD36 were similar or lower, respectively, in cancer tissues comparing to normal mammary gland tissues (Supplementary Fig. 4). Additionally, we stratified the results of gene expression analysis by cancer stage (Supplementary Table S4). We did not find any significant differences between mRNA levels in patients with I and II grade of BC (Supplementary Table S4).

Finally, we decided to investigate whether the accumulation of VLCFAs was restricted to only cancer tissue and analyzed the FA content of breast adipose tissues from both patients and control subjects. GC-MS analysis revealed that breast fat depots from healthy control subjects were surprisingly richer in VLCFAs than those of BC patients (Supplementary Table S5).

### Analysis of circulating FAs

Basic lipid profile analysis revealed that BC patients (Table 1) were characterized by lower high-density lipoprotein (HDL) cholesterol but showed no differences from healthy control subjects in serum low-density (LDL) cholesterol, total cholesterol or triglyceride (TG) concentrations. The extent of lipid peroxidation and NEFA concentrations in the serum of BC patients was higher compared to serum of control subjects (Table 1). Patients, had fewer indicators of oxidative damage to proteins (protein carbonyls), whereas higher TBARS concentrations which indicate oxidative damage to lipids (Table 1). We found no correlations between these markers and VLCFAs levels. More detailed analysis of serum lipidome of patients and controls by mass spectrometry allowed us to identify large differences in FA profiles between the two groups (Table 3). The BC patients were characterized by significantly lower content of n-3 PUFAs with 22-carbons, i.e. n-3 DPA and the most abundant DHA, representing the largest difference. There were no significant differences in monounsaturated FAs (MUFAs) and odd-chain FAs (OCFAs) between BC patients and control subjects, but the patients had higher serum levels of *iso-* and *anteiso-*BCFAs. The most striking feature of the patient serum was the presence of higher levels of some even-chain VLCFAs (26:0 and 28:0), but other VLCFAs did not show this tendency. Group differences in serum fatty acid levels were also assessed using ANCOVA with BMI as a covariate, the significance of group differences did not persist after the Benjamini–Hochberg procedure. Regression coefficients between BMI and serum fatty acids from ANCOVA models including group as a covariate are included in Supplementary Table S6. The strongest associations of FA content with BMI were observed for PUFAs (slope − 0.349, adjusted p-value 0.029), there was significant but small association between BMI and even-chained saturated VLCFAs (slope − 0.014, adjusted p-value 0.029). The stratification of the data by cancer stage (Supplementary Table S7) or histopathologic type (Supplementary Table S8) revealed no significant differences, except for significantly higher 24:0 in sera of BC patients with luminal B than those with luminal A histopathological type (Supplementary Table S8). Surprisingly, the BC patients had higher levels of lauric acid (12:0) and myristic acid (14:0). Of most abundant saturated FAs (SFAs), the level of palmitic acid (16:0) did not differ from the serum of control group, while the level of stearic acid (18:0) was slightly higher in the patients.

**Table 3:** Fatty acid content [%] in serum from serum of cancer patients and healthy control subjects

	Patients’ serum (*n* = 53)	Control serum (*n* = 23)	*p*-value without adjustment for BMI	raw *p* – value adjusted for BMI	BH corrected *p*-value adjusted for BMI
10:0	0.012 ± 0.008	0.009 ± 0.005	0.155	0.149	0.316
12:0	0.139 ± 0.101	0.070 ± 0.025	0.003*	0.001	0.035
18:0	8.12 ± 2.28	7.48 ± 1.39	0.008*	0.020	0.100
Even-chained VLCFAs	0.586 ± 0.030	0.560 ± 0.048	0.275	0.577	0.776
20:0	0.151 ± 0.073	0.137 ± 0.060	0.154	0.359	0.590
22:0	0.204 ± 0.078	0.189 ± 0.088	0.103	0.358	0.590
24:0	0.199 ± 0.079	0.213 ± 0.087	0.768	0.397	0.619
26:0	0.026 ± 0.013	0.019 ± 0.010	0.020*	0.013*	0.100
28:0	0.010 ± 0.007	0.003 ± 0.004	< 0.001*	0.019*	0.100
Other ECFA	1.18 ± 0.578	0.85 ± 0.241	0.004*	0.699	0.877
Sum of ECFA	33.0 ± 8.80	31.7 ± 3.25	0.009	0.045*	0.150
11:0	0.005 ± 0.004	traces	< 0.001*	< 0.001*	< 0.001*
13:0	0.013 ± 0.006	0.012 ± 0.004	0.476	0.418	0.631
Odd-chained VLCFAs	0.114 ± 0.006	0.115 ± 0.010	0.680	0.878	0.928
21:0	0.020 ± 0.009	0.020 ± 0.010	0.797	0.930	0.956
23:0	0.081 ± 0.034	0.080 ± 0.034	0.707	0.965	0.965
25:0	0.013 ± 0.007	0.015 ± 0.011	0.653	0.334	0.589
Other OCFA	0.131 ± 0.005	0.127 ± 0.011	0.387	0.457	0.651
Sum of OCFA	0.707 ± 0.223	0.699 ± 0.153	0.469	0.789	0.922
*iso* 15:0	0.023 ± 0.011	0.019 ± 0.010	0.059	0.102	0.237
*iso* 16:0	0.048 ± 0.022	0.040 ± 0.014	0.066	0.057	0.168
*iso* 17:0	0.098 ± 0.042	0.080 ± 0.031	0.036*	0.032*	0.129
*iso* 22:0	0.006 ± 0.005	0.005 ± 0.005	0.040*	0.451	0.655
Other *iso* BCFA	0.010 ± 0.004	0.007 ± 0.006	0.005*	0.007*	0.064
Sum of *iso* BCFA	0.185 ± 0.073	0.151 ± 0.055	0.014*	0.016*	0.100
anteiso 17:0	0.086 ± 0.037	0.069 ± 0.025	0.025*	0.022*	0.100
anteiso 19:0	0.035 ± 0.015	0.028 ± 0.013	0.023*	0.026*	0.114
anteiso 23:0	0.008 ± 0.005	traces	< 0.001*	0.002*	0.035*
Other *anteiso* BCFA	0.039 ± 0.021	0.036 ± 0.023	0.294	0.507	0.695
Sum of *anteiso* BCFA	0.167 ± 0.066	0.136 ± 0.054	0.007*	0.018*	0.100
Sum of BCFA	0.367 ± 0.140	0.304 ± 0.114	0.012*	0.020*	0.100
Saturated VLCFAs	0.670 ± 0.029	0.675 ± 0.058	0.295	0.658	0.840
Sum of SFA	34.1 ± 9.09	32.7 ± 3.24	0.010*	0.040*	0.144
19:1	0.011 ± 0.005	0.012 ± 0.006	0.887	0.920	0.956
Monounsaturated VLCFAs	0.466 ± 0.024	0.469 ± 0.023	0.769	0.826	0.922
20:1	0.182 ± 0.106	0.165 ± 0.045	0.946	0.432	0.639
22:1	0.028 ± 0.056	0.019 ± 0.007	0.840	0.491	0.686
24:1	0.256 ± 0.121	0.274 ± 0.101	0.479	0.474	0.675
Other MUFA	31.6 ± 0.562	29.9 ± 0.678	0.153	0.071	0.190
Sum of MUFA	32.1 ± 9.16	30.3 ± 3.24	0.149	0.072	0.190
AdA 22:4 n-6	0.113 ± 0.044	0.131 ± 0.036	0.040*	0.041*	0.144
Other PUFA n-6	31.2 ± 0.672	33.2 ± 0.990	0.094	0.059	0.168
Sum of PUFA n-6	31.1 ± 9.31	33.4 ± 4.75	0.068	0.057	0.168
ETA 20:4 n-3	0.061 ± 0.032	0.076 ± 0.022	0.014*	0.033*	0.129
DHA 22:6 n-3	1.35 ± 0.625	1.86 ± 0.877	0.012*	0.002*	0.035*
n-3 DPA 22:5 n-3	0.328 ± 0.127	0.430 ± 0.118	< 0.001*	< 0.001*	0.007*
Other PUFA n-3	0.990 ± 0.064	1.20 ± 0.111	0.074	0.088	0.217
Sum of PUFA n-3	2.73 ± 1.22	3.57 ± 1.44	0.011*	0.005*	0.056
Sum of PUFA	33.9 ± 10.2	36.9 ± 5.04	0.023*	0.018*	0.100

Values are mean ± SD; BH, Benjamini–Hochberg; ND, not detected; traces indicated that diagnostic ions were present, but the signal was too small to be integrated. * denotes significant p-value. For FA abbreviations see Table 2

Other ECFA = 14:0

Other OCFA = 15:0 + 17: 0 + 19:0

Other iso BCFA = iso 14:0

Other anteiso BCFA = anteiso 15:0

Other MUFA = 14:1 + 16:1 + 17:1

Other PUFA n-6 = 16:2 n-6 + 18:2 n-6 + 20:4 n-6 + 20:3 n-6 + 20:2 n-6 + 22:5 n-6

Other PUFA n-3 = 18:3 n-3 + 20:5 n-3

**Fig. 3:**
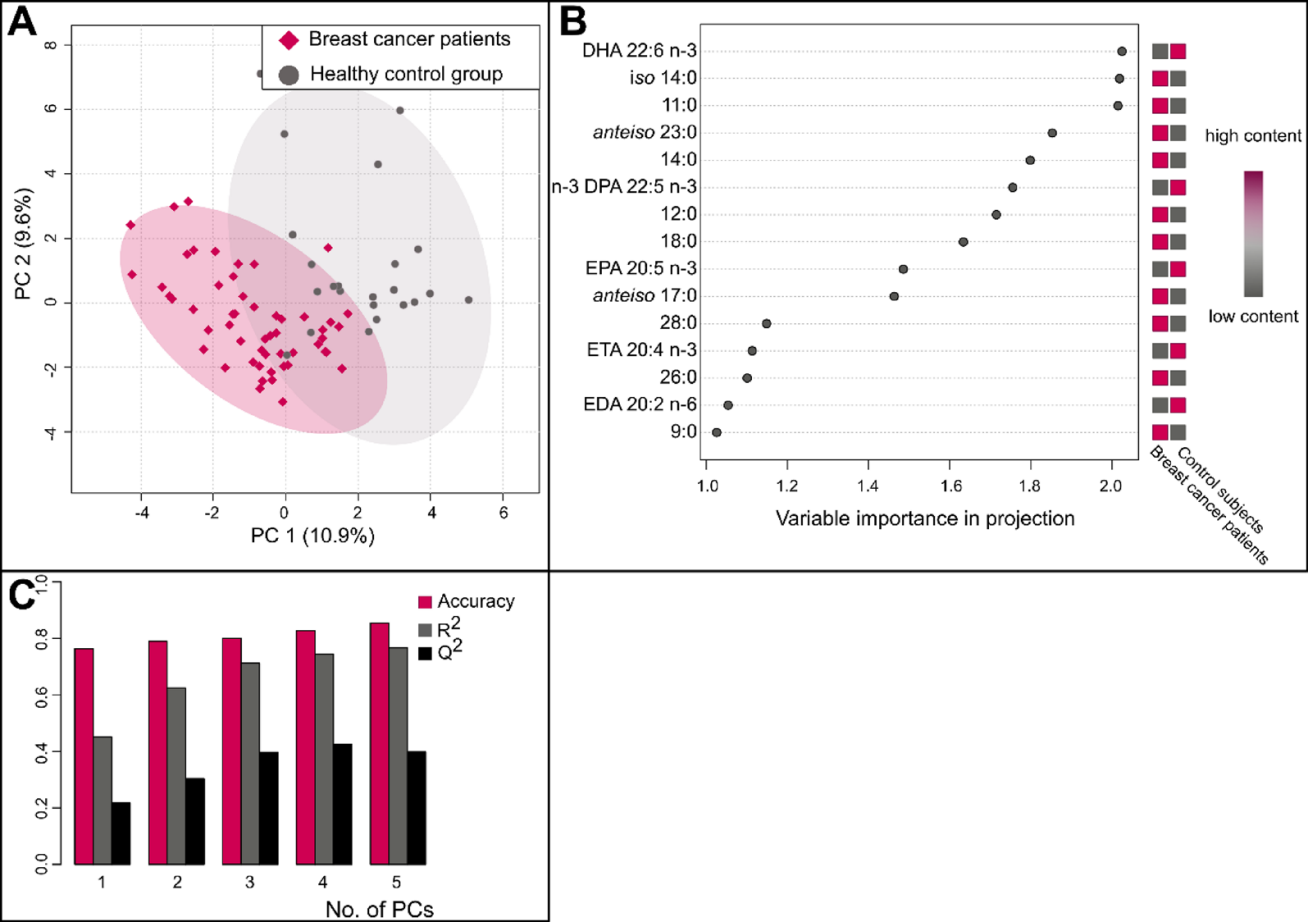
Discriminant PLS-DA model constructed from FA content in serum of breast cancer patients and control subjects. (**A**) – PLS-DA projection, the ellipse represents the Hotelling T^2^ with 95% confidence interval, analysis included all individual FAs adjusted for BMI, first two latent variables explain 20.5% of variance; (**B**) – variable importance in projection (VIP) scores, showing the FAs contribution to the model, threshold for importance was VIP > 1; (**C**) – cross validation results of model performance, method used was 5-fold CV, p was < 0.05 from model cross-validation, and Hotteling. R^2^, measure of model fit to the original data; Q^2^, predictive relevance of the model

To investigate whether the total serum FA profile can be used to differentiate between BC patients and healthy control subjects and to identify the FAs that contribute most to these differences, supervised PLS-DA analysis was performed. The score plot in Fig. 3A shows that clustering of BC patients was achieved in this model. VLCFAs 26:0 was among the top 15 most important diagnostic features according to the variable importance in projection (VIP) scores (Fig. 3B) and there were low individual ROC areas under the curve for VLCFAs (Supplementary Figure S5). Unfortunately, the PLS-DA model performed poorly in its predictive ability (Q^2^ values below 0.8), suggesting that serum FA content is not sufficient to determine the presence of BC.

## Discussion

Lipid metabolism has been recognized as an important player in cancer cell specific biology, and a considerable number of studies have been devoted to the role of *de novo* FA synthesis by fatty acid synthase (FASN), an enzyme that is overexpressed in malignant breast tumors [18]. However, recent evidence shows that the attenuated endogenous synthesis can be compensated by lipid uptake [19], so that inhibition of FASN has no effect on FA elongation products, as shown by Chu et al. [4]. Many early studies on the FA composition of BC tissues omitted the saturated VLCFAs because their analysis requires longer chromatographic separation time [20–22]. The major finding of our study is the identification of a much higher proportion of VLCFAs, especially VLCFAs with saturated acyl chains, in cancer tissue than in normal breast tissue from BC patients. Previous GC-MS studies of serum FAs in patients with invasive ductal carcinoma, showed an accumulation of SFAs, including 22:0 [23]. Tan et al. [14] similarly reported increased concentrations of 22:0 and 24:0 in the fraction of free FAs in the serum of BC patients. In BC tissue, Yamashita et al. [5] detected higher levels of 22:0 and 24:0 in BC tissue compared to the corresponding normal breast tissue, but did not find increased content of saturated VLCFAs with more than 24 carbons. In contrast, in our analysis we found a quantifiable 26:0 content in cancerous tissue, whereas this FA was only present in trace amounts in normal breast tissue from breast cancer patients. Similarly, in our study, C > 24:0 FAs were more numerous in the serum of patients. The accumulation of cerotic acid in cancer tissue and patient serum has previously been associated with colorectal cancer [3, 6]. Considering the differences between our study and that of Yamashita et al. [5] with respect to the content of saturated VLCFAs in BC, a possible explanation could be population differences, as one study was conducted in Poland and the other in Japan. However, both studies indicate an increased level of VLCFA in BC tissue. Physiological conditions, dietary habits and individual metabolism are some of the variables that could affect the relative contribution of endogenous synthesis and dietary intake to VLCFA levels in the body. Studies on dietary management in adrenoleukodystrophy have shown that the intake of 26:0 may vary depending on type of products consumed [24], the consumption of which could actually alter the blood levels of this FA [25].

The excessive amounts of saturated VLCFAs in our study were a feature of the tumor tissue but not of the adipose tissue in patients’ breasts. Increased levels of VLCFA in BC cancer tissue could be explained either by increased synthesis of VLCFAs by cancer cells or by increased uptake from outside the tumor. To test the contribution of in situ elongation to the levels of VLCFA in BC tissue we analyzed ELOVL1 mRNA and protein levels in BC cancer and normal mammary gland tissues from breast cancer patients. However, we found no evidence that this enzyme is more abundant in tumor tissue. Higher expression of this enzyme was observed in colorectal cancer [6] and hepatocellular carcinoma [26]. This result is in contrast to an earlier analysis [5] showing ELOVL1 overexpression in triple-negative breast cancer (TNBC). However, it should be noted that the TNBC stained more strongly for ELOVL1 than hormone-positive, HER2-negative cancer tissue, which is mainly represented in our analysis (luminal A and luminal B subtypes). Hilvo et al. [27] also reported no overexpression of ELOVL1 mRNA in breast cancer tissues, although increased levels of saturated and monounsaturated VLCFAs were present in phospholipids of cancer cells. Nevertheless, the gene silencing results indicated that ELOVL1 is involved in cancer cell viability [27]. The lack of increased expression of other ELOVL family enzymes in cancer tissue compared to normal gland tissue from BC patients also suggests that other isoforms of ELOVL are not responsible for increased VLCFA in BC tissue. Our analysis showed that although the breast cancer tissue had higher levels of VLCFAs, this was not true for the patients’ breast adipose tissue, where the level of VLCFAs was lower than in the breast adipose tissue from control subjects. Using co-cultures of breast cancer cells with adipocytes, Yang et al. [28] demonstrated that the progression of breast cancer is dependent on adipocyte-derived lipids. The adipocytes located near the tumor (so-called cancer-associated adipocytes - CAAs) have an altered metabolism and provide various metabolites to the cancer cells, including fatty acids [29]. It could therefore be that the VLCFAs might originate in part from breast adipose tissue (where their level decreases at the same time), but this needs to be further confirmed by examining the expression of elongases in breast fat. In our study, the adipose tissue from BC patients was not directly located by the tumor, but the signaling molecules released from the cancer tissue could also affect adipocytes throughout the whole breast with the tumor. Surprisingly, we found a lower mRNA level of CD36 in cancer tissue than in normal mammary gland tissue from BC patients and no difference in FATP2. However, it could be considered that CD36 can transport FA inside and outside the cells, and it cannot be excluded that normal mammary gland tissue cells also supply cancer cells with VLCFA. In such a situation, CD36 and FATP could be overexpressed both in normal tissue surrounding the tumor (to export VLCFA) and in tumor tissue (to import VLCFA). However, to address this issue mRNA mammary gland tissue from healthy subjects should be examined, but unfortunately we do not have such tissue to compare it to tissues from BC patients. Another possible reason for increased VLCFA in BC tissue could be impaired VLCFA oxidation. VLCFA are mainly oxidized in the peroxisomes [10], so perhaps reduced peroxisomal β-oxidation could be involved, but we have not investigated this aspect so far. It should be noted that impaired insulin sensitivity (which is a case in our BC patients – see elevated concentrations of insulin and c-peptide as well as HOMA values in Table 1) results in impaired peroxisome metabolism [30]. This problem requires further studies. Certainly, the involvement of ELOVL1 in breast cancer metabolism need to be further elucidated to determine the feasibility of therapeutic approaches against this enzyme.

The effects of increased levels of VLCFAs and VLCFAs metabolism on breast cancer biology remain unclear, although evidence from studies of other pathologies suggests that these FAs are involved in many cancer hallmarks. In pancreatic cancer, accumulation of long-chain FAs and VLCFAs has been shown to promote dysfunction of tumor surveilling T-cells [31]. Lipids containing VLCFAs with 24 carbon chains at higher than physiological doses, were shown to permeate and disrupt liposome membranes and play an active role in necroptosis [32], a pathway that contributes to cancer metastasis and T-cells death [33]. However, the above mechanisms of VLCFA actions need to be verified in BC cells. The levels of VLCFA in breast cancer cells are relatively low, so it is unlikely that they play an important role in cellular energy metabolism. Nonetheless, the presence of VLCFA in cell membrane affect their properties including permeability [12], and thus increased level of VCFA in the membrane of cancer cells could be a defense mechanism against agents with anticancer properties, including drugs. However, this issue requires further studies. In neurodegenerative cell models VLCFAs contribute to the toxicity of reactive oxygen species [34]. However, we did not find an association between common markers of oxidative stress (protein carbonyls and TBARS) and VLCFAs levels in BC tissue (not shown). Also noteworthy is the relationship between VCLFAs and cell organelles that exhibit aberrant metabolism associated with cancer, e.g. peroxisomes [10] and the endoplasmic reticulum [35]. Exposure of fibroblasts from adrenoleukodystrophy to methylated 22:0, 24:0 and 26:0 leads to an endoplasmic reticulum stress [9]. In turn, the severity of peroxisomal disorders can be correlated with 26:0 abundance, with VLCFAs thought to contribute to mitochondrial membrane disruption [36].

To date, more attention has been focused on investigating the involvement of polyunsaturated VLCFAs in the pathogenesis of breast cancer [37]. PUFA-specific ELOVL5 downregulation in breast cancer patients, particularly in ER + tumors, has been identified as a factor contributing to cancer proliferation and progression, with its knockdown shown to promote metastasis [38, 39]. In turn downregulation of ELOVL2 by DNA methylation has been found to be associated with tamoxifen-resistant BC [40]. Not surprisingly, our analysis indicates that long-chain PUFAs, namely DHA, EPA and DPA n-3, are the major contributors to the overall difference in FA profiles between patients and controls. Lower levels of serum n-3 PUFA are consistent with our findings in colorectal cancer (CRC) patients in whom serum PUFA were decreased [41]. Our previous in vitro studies suggested that this may be related to the preferential uptake of PUFA from the blood of CRC patients [41]. This could also be a case in BC, as the level of n-3 PUFA in BC tissue was higher in the normal mammary gland from BC patients. The results of this study clearly suggest that saturated VLCFAs also serve as differentiating factors and merit of further research into the role they may play in breast cancer metabolism.

The major strength of this study is the comprehensive analysis of FA, including VLCFAs, in both breast cancer tissue and serum. The novel aspect of considering VLCFA content in breast adipose tissue is of additional value to the study. The study includes a limited sample size, so there is a possible influence of population differences on FA profiles. The number of serum samples (53 vs. 23 controls) and adipose tissue samples (34 vs. 10) is unbalanced and low in the control group. There is evidence that FA may differ according to BC subtype [5], but comparison of luminal A, luminal B and luminal B HER2 + showed no significant differences in VLCFA between these subgroups of patients. Unfortunately, we were not able to recruit a sufficient number of TNBC patients to include this comparison. Another limitation of the study could be the lack of analysis on the influence of diet of the BC patients and the control group on VLCFA levels. However, the short period of time (14 days) for which changes in the diet were recommended in the preoperative phase is unlikely to significantly affect the VLCFA levels in blood [24]. In addition, the exact sources contributing to saturated VLCFAs levels in breast cancer need to be further elucidated in experiments that consider both the expression and activity of specific elongases in relevant tissues and possible exogenous sources of these FAs. Technical limitations include the lack of separation of total lipids into lipid classes, which is why we present the results of FA composition of total lipids in the tissues and serum examined.

## Conclusions

This study investigated the FA composition of tissue and serum of BC patients and found a significantly higher proportion of saturated VLCFAs in cancer tissue and patient serum compared to normal mammary gland tissue from breast cancer patients and serum from healthy subjects. In contrast to previous findings, the results of the analysis of ELOVL1 expression suggest that these VLCFAs could originate from lipid uptake from outside the tumor and are not synthesized in situ. Given the recent progress in understanding the contribution of VLCFAs to metabolic disorders in various diseases these results emphasize the need for further research on their specific contributions to breast cancer biology, which could help in determining potential therapeutic targets.

## Supplementary Information

Supplementary Material 1. The additional files include: Supplementary Table S1. Comparison of VLCFAs groups content in cancer tissue stratified for stage, Supplementary Table S2. Comparison of VLCFAs groups content in cancer tissue stratified for histopathological type, Supplementary Table S3 Fatty acid content [%] in fatty tissues from breasts of cancer patients and healthy control subjects and Supplementary Figure S1 Representative H&E-stained images of cancer tissueand normal fibroglandular tissue from breast cancer patient. Supplementary Table S4. Comparative analysis of gene expression profiles in tumor tissues of patients of cancer stages I and II, Supplementary Table S5 provides primer sequences used to assess gene expression by RT-PCR. Supplementary Figure S2 Oxidative stress parameters and NEFA content in tissues from breast cancer patients. Supplementary Figure S3 ROC curves and corresponding boxplots of serum content of different FA groups with very long chains, Supplementary Table S6. BMI-associated effects on serum fatty acid levels estimated using ANCOVA, Supplementary Table S7 Comparison of VLCFAs groups content in serum stratified for stage, Supplementary Table S8. Comparison of VLCFAs groups content in serum stratified for histopathological type.

## Data Availability

The original data of fatty acid composition of serum and tissues are available in RepOD as part of the project titled “Alterations of fatty acid profiles of different lipid classes in breast cancer - evaluation of their role and diagnostic significance” at 10.18150/MHI1FZ.
