# Supplementary material for: Very long-chain fatty acids accumulate in breast cancer tissue and serum
Source: Cancer Cell Int. 2025 Aug 4;25:296. doi: 10.1186/s12935-025-03928-2 (PMC12320370; doi:10.1186/s12935-025-03928-2)
Supplement: Supplementary file 1 — Supplementary Material 1. The additional files include: Supplementary Table S1. Comparison of VLCFAs groups content in cancer tissue stratified for stage, Supplementary Table S2. Comparison of VLCFAs groups content in cancer tissue stratified for histopathological type, Supplementary Table S3 Fatty acid content [%] in fatty tissues from breasts of cancer patients and healthy control subjects and Supplementary Figure S1 Representative H&E-stained images of cancer tissueand normal fibroglandular tissue from breast cancer patient. Supplementary Table S4. Comparative analysis of gene expression profiles in tumor tissues of patients of cancer stages I and II, Supplementary Table S5 provides primer sequences used to assess gene expression by RT-PCR. Supplementary Figure S2 Oxidative stress parameters and NEFA content in tissues from breast cancer patients. Supplementary Figure S3 ROC curves and corresponding boxplots of serum content of different FA groups with very long chains, Supplementary Table S6. BMI-associated effects on serum fatty acid levels estimated using ANCOVA, Supplementary Table S7 Comparison of VLCFAs groups content in serum stratified for stage, Supplementary Table S8. Comparison of VLCFAs groups content in serum stratified for histopathological type. [file 12935_2025_3928_MOESM1_ESM.docx]

**Supplementary material for “Very long-chain fatty acids accumulate in breast cancer tissue and serum”.**

Alicja Pakiet, Michalina Ciosek, Oliwia Lange, Katarzyna Duzowska, Agata Janczy, Małgorzata Kapusta, Yelyzaveta Razghonova, Marcin Ekman, Anna Abacjew-Chmyłko, Paweł Kabata & Adriana Mika


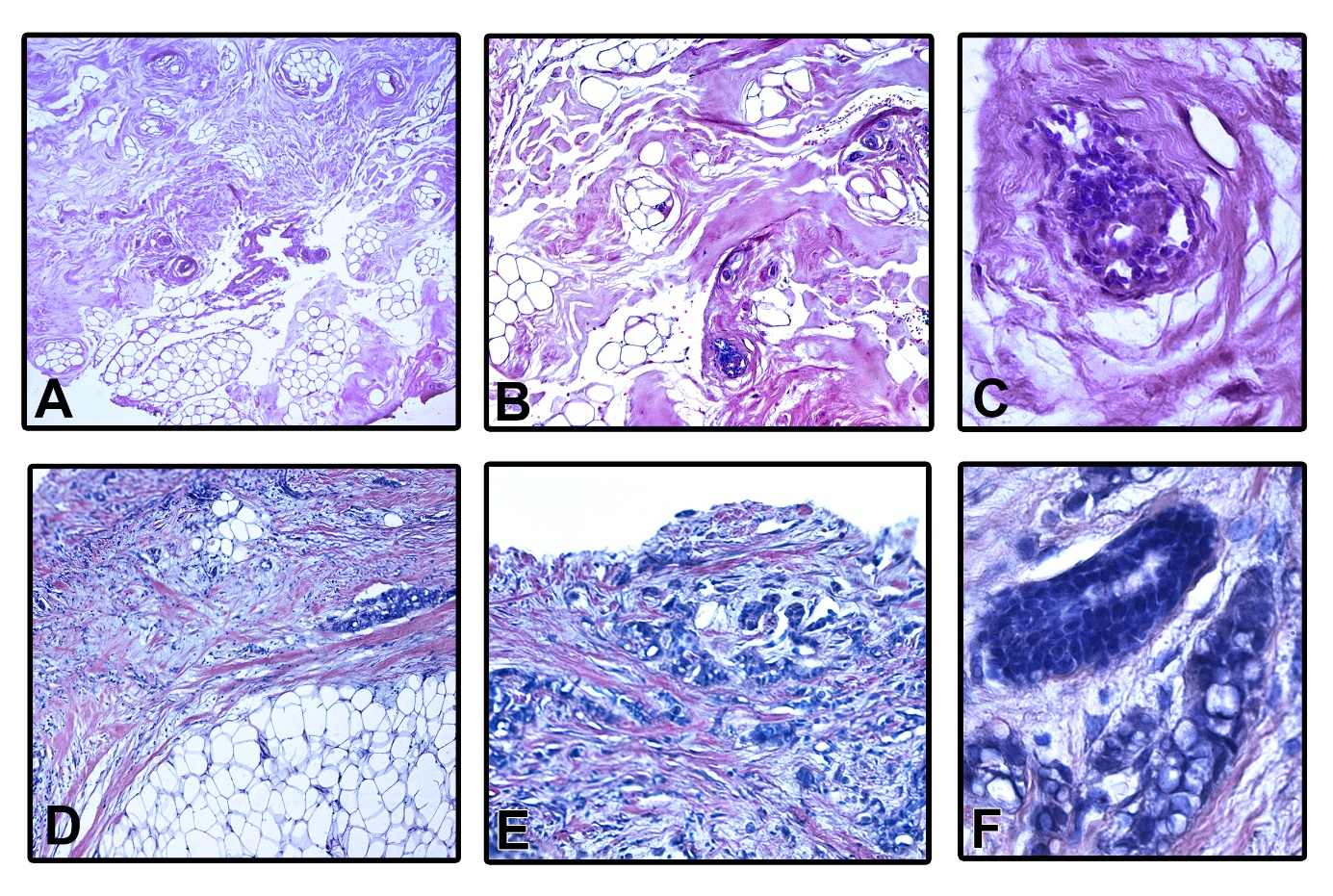


Supplementary Figure S1 Representative H&E-stained images of cancer tissue (A-C) and normal fibroglandular tissue from breast cancer patient (D-F).

Panels A and D at 40x, B and E at 100x, C and F at 400x magnification.

**Supplementary Table S1** Primer sequences used to assess gene expression by RT-PCR

| **Target genes** | **Sequence R** | **Sequence F** |
| --- | --- | --- |
| ***Cyclophilin A*** | TCGAGTTGTCCACAGTCA | CGTCTCCTTTGAGCTGT |
| ***ELOVL1*** | CTGGGAGATGTGCAGTGAGA | CTGTGGCACAACCCTACCTT |
| ***ELOVL2*** | CCCAGCCATATTGAGAGCAGATA | ATGTTTGGACCGCGAGATTCT |
| ***ELOVL3*** | GTAGCACAGTCCCCATAATGCC | CTACCTGGTTCTCATCGCTGTG |
| ***ELOVL4*** | CACACGCTTATCTGCGATGG | GAGCCGGGTAGTGTCCTAAAC |
| ***ELOVL5*** | ACCAGAGGACACGGATAATCTT | TAACAGGAGTATGGGAAGGCA |
| ***ELOVL6*** | GGCAACCATGTCTTTGTAGCA | CTAAGCAAAGCACCCGAACT |
| ***ELOVL7*** | AGGACATGAGGAGCCAATCTT | GCCTTCAGTGATCTTACATCGAG |
| ***CD36*** | GAACTGCAATACCTGGCTTTTCTC | AAGTCACTGCGACATGATTAATGG |
| ***FATP2*** | CCTCGTAAGCCATTTCCCAGT | CTCTTGCCTTGCGGACTAAAT |

*Note:* ELOVL1,2,3,4,5,6,7 – very long chain fatty acid elongase; CD36 – Fatty Acid Translocase; FATP2 – Fatty Acid Transport Protein 2.

**
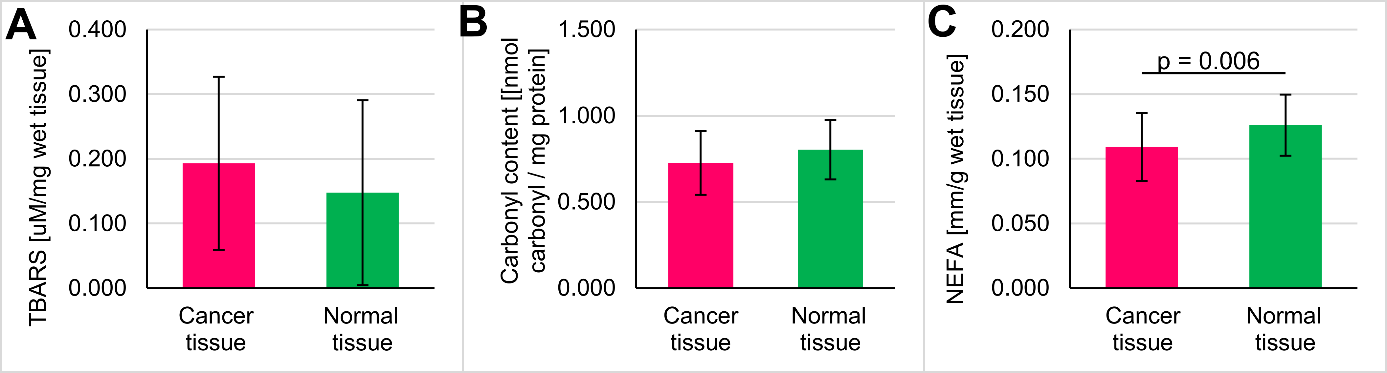
**

Supplementary Figure S2 Oxidative stress parameters and NEFA content in tissues from breast cancer patients.


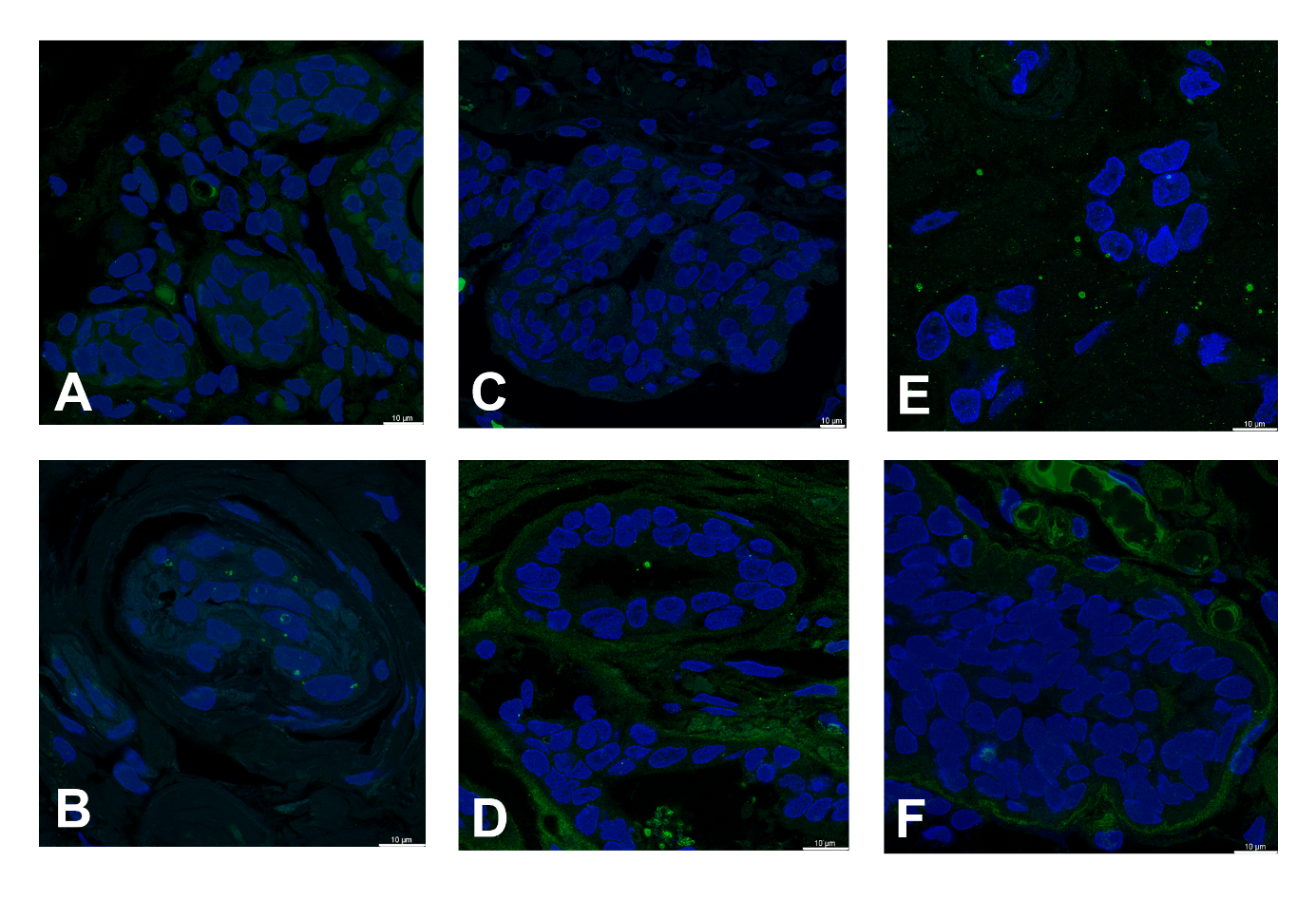


Supplementary Figure S3 Negative controls for immunoflurescent detection of ELOVL1 created by omitting the primary antibody step. Representative images of cancer tissue (A, C, and E) and complementary normal tissue breast with mammary ducts from cancer patients (B, D, and F).

All panels at 630x magnification.

Supplementary Table S2. Comparison of VLCFAs groups content in cancer tissue stratified for stage

| **Cancer tissue** | **Stage I**  **(n = 21)** | **Stage II and III**  **(n = 28)** | **p-value** |
| --- | --- | --- | --- |
| **Even-chained VLCFAs ≥ 20:0** | 0.236 ± 0.018 | 0.212 ± 0.026 | 0.082 |
| **20:0** | 0.146 ± 0.01 | 0.134 ± 0.012 | 0.451 |
| **22:0** | 0.04 ± 0.004 | 0.038 ± 0.009 | 0.879 |
| **24:0** | 0.043 ± 0.007 | 0.035 ± 0.006 | 0.357 |
| **26:0** | 0.007 ± 0.002 | 0.006 ± 0.001 | 0.627 |
| **Odd-chained VLCFAs ≥ 21:0** | 0.026 ± 0.004 | 0.023 ± 0.004 | 0.633 |
| **21:0** | 0.009 ± 0.002 | 0.009 ± 0.001 | 0.991 |
| **23:0** | 0.013 ± 0.002 | 0.012 ± 0.002 | 0.759 |
| **25:0** | 0.004 ± 0.001 | 0.002 ± 0.001 | 0.285 |
| **Saturated VLCFAs** | 0.262 ± 0.022 | 0.235 ± 0.03 | 0.115 |
| **Monounsaturated VLCFAs ≥ 20:1** | 1.03 ± 0.068 | 0.995 ± 0.045 | 0.668 |
| **20:1** | 0.939 ± 0.062 | 0.921 ± 0.039 | 0.804 |
| **22:1** | 0.045 ± 0.005 | 0.042 ± 0.005 | 0.694 |
| **24:1** | 0.044 ± 0.018 | 0.031 ± 0.007 | 0.471 |

Values are mean ± SD

Supplementary Table S3. Comparison of VLCFAs groups content in cancer tissue stratified for histopathological type

| **Cancer tissue** | **Luminal A**  **(n = 20)** | **Luminal B**  **(n = 22)** | **Luminal B HER2+**  **(n = 5)** | **p-value**  **Luminal A vs Luminal B** | **p-value**  **Luminal A vs Luminal B HER2+** | **p-value**  **Luminal B vs Luminal B HER2+** |
| --- | --- | --- | --- | --- | --- | --- |
| **Even-chained VLCFAs ≥ 20:0** | 0.209 ± 0.025 | 0.208 ± 0.024 | 0.382 ± 0.051 | 0.989 | 0.393 | 0.394 |
| **20:0** | 0.137 ± 0.012 | 0.132 ± 0.011 | 0.206 ± 0.025 | 0.615 | 0.376 | 0.228 |
| **22:0** | 0.034 ± 0.008 | 0.035 ± 0.008 | 0.086 ± 0.016 | 0.742 | 0.392 | 0.510 |
| **24:0** | 0.036 ± 0.017 | 0.035 ± 0.006 | 0.076 ± 0.014 | 0.463 | 0.286 | 0.536 |
| **26:0** | 0.003 ± 0.003 | 0.007 ± 0.002 | 0.014 ± 0.004 | 0.008 | 0.238 | 0.635 |
| **Odd-chained VLCFAs ≥ 21:0** | 0.021 ± 0.005 | 0.023 ± 0.005 | 0.041 ± 0.01 | 0.279 | 0.477 | 0.966 |
| **21:0** | 0.008 ± 0.002 | 0.010 ± 0.002 | 0.008 ± 0.003 | 0.120 | 0.705 | 0.176 |
| **23:0** | 0.013 ± 0.003 | 0.010 ± 0.003 | 0.025 ± 0.005 | 0.856 | 0.546 | 0.470 |
| **25:0** | 0.001 ± 0.001 | 0.003 ± 0.001 | 0.008 ± 0.002 | 0.039* | 0.117 | 0.769 |
| **Saturated VLCFAs** | 0.230 ± 0.03 | 0.231 ± 0.028 | 0.423 ± 0.059 | 0.811 | 0.372 | 0.452 |
| **Monounsaturated VLCFAs ≥ 20:1** | 1.07 ± 0.065 | 0.991 ± 0.062 | 1.07 ± 0.131 | 0.555 | 0.675 | 0.956 |
| **20:1** | 0.988 ± 0.058 | 0.917 ± 0.056 | 0.956 ± 0.117 | 0.764 | 0.602 | 0.734 |
| **22:1** | 0.046 ± 0.006 | 0.041 ± 0.006 | 0.048 ± 0.012 | 0.329 | 0.816 | 0.708 |
| **24:1** | 0.039 ± 0.014 | 0.032 ± 0.014 | 0.062 ± 0.029 | 0.151 | 0.287 | 0.858 |

Values are mean ± SD, * indicate p-values < 0.05


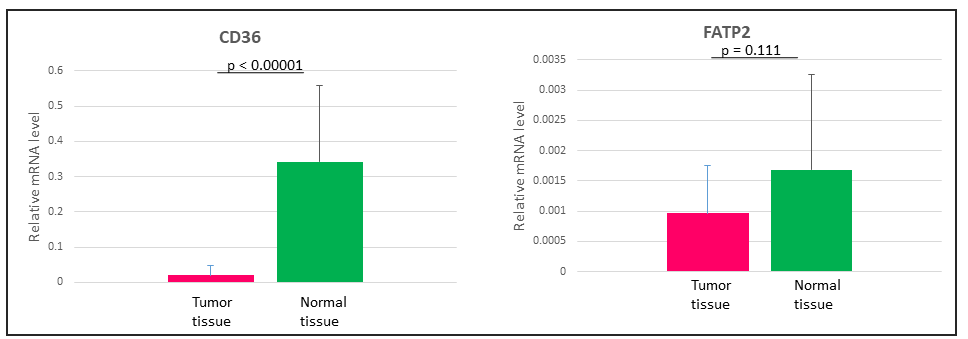


Supplementary Figure S4. The expression of CD36 and FATP2 in cancer tissue and normal breast gland tissue from breast cancer patients.

*Note:* CD36 – Fatty Acid Translocase; FATP2 – Fatty Acid Transport Protein 2.

Supplementary Table S4. Comparative analysis of gene expression profiles in tumor tissues of patients of cancer stages I and II

|  | **Stage I (cancer tissue)**  **(n = 12)** | **Stage II (cancer tissue)**  **(n = 7)** | **p-value** |
| --- | --- | --- | --- |
| **ELOVL1** | 0.008 ± 0.004 | 0.013 ± 0.005 | 0.490 |
| **ELOVL2** | 0.0013 ± 0.0004 | 0.0004 ± 0.00013 | 0.186 |
| **ELOVL3** | 0.0007 ± 0.0002 | 0.001 ± 0.0004 | 0.212 |
| **ELOVL4** | 0.00005 ± 1E-05 | 0.0001 ± 3E-05 | 0.115 |
| **ELOVL5** | 0.006 ± 0.002 | 0.005 ± 0.001 | 0.627 |
| **ELOVL6** | 0.0004 ± 9E-05 | 0.0005 ± 0.0001 | 0.704 |
| **ELOVL7** | 0.0003 ± 8E-05 | 0.0003 ± 7.69E-05 | 0.638 |
| **CD36** | 0.019 ± 0.006 | 0.023 ± 0.009 | 0.766 |
| **FATP2** | 0.0007 ± 0.0002 | 0.0014 ± 0.0003 | 0.117 |

Values are mean ± SEM. This analysis included 19 patients, because the RNA isolated from cancer or normal tissue or both from other patients did not rich enough high quality of RQI above 7 out of 10 in the Experion RNA integrity analysis (see Methods). * indicate p-values < 0.05

Supplementary Table S5 Fatty acid content [%] in fatty tissues from breasts of cancer patients and healthy control subjects

|  | **BC patients**  **(n = 29)** | **Control (n=10)** | p-value |
| --- | --- | --- | --- |
| **10:0** | 0.008 ± 0.006 | 0.018 ± 0.012 | 0.010* |
| **12:0** | 0.345 ± 0.194 | 0.374 ± 0.182 | 0.499 |
| **14:0** | 2.93 ± 1.10 | 3.01 ± 1.19 | 0.735 |
| **16:0** | 22.8 ± 7.74 | 22.1 ± 6.94 | 0.261 |
| **18:0** | 5.18 ± 1.98 | 5.00 ± 1.96 | 0.669 |
| **Even-chained VLCFAs** | 0.205 ± 0.021 | 0.25 ± 0.030 | 0.029* |
| **20:0** | 0.168 ± 0.088 | 0.186 ± 0.068 | 0.127 |
| **22:0** | 0.023 ± 0.019 | 0.035 ± 0.017 | 0.010* |
| **24:0** | 0.013 ± 0.010 | 0.028 ± 0.013 | < 0.001* |
| **26:0** | traces | 0.003 ± 0.006 | 0.127 |
| **Sum of ECFA** | 31.5 ± 10.7 | 30.7 ± 9.83 | 0.463 |
| **11:0** | 0.002 ± 0.002 | 0.007 ± 0.005 | 0.040* |
| **13:0** | 0.012 ± 0.005 | 0.018 ± 0.007 | 0.003* |
| **15:0** | 0.304 ± 0.122 | 0.290 ± 0.126 | 0.630 |
| **17:0** | 0.242 ± 0.091 | 0.219 ± 0.082 | 0.190 |
| **19:0** | 0.022 ± 0.010 | 0.024 ± 0.011 | 0.592 |
| **Odd-chained VLCFAs** | 0.009 ± 0.001 | 0.013 ± 0.003 | 0.220 |
| **21:0** | 0.007 ± 0.005 | 0.008 ± 0.006 | 0.431 |
| **23:0** | 0.004 ± 0.004 | 0.008 ± 0.005 | 0.187 |
| **25:0** | traces | ND | - |
| **Sum of OCFA** | 0.592 ± 0.226 | 0.570 ± 0.230 | 0.653 |
| **iso 14:0** | 0.014 ± 0.007 | 0.020 ± 0.011 | 0.085 |
| **iso 15:0** | 0.037 ± 0.017 | 0.040 ± 0.020 | 0.537 |
| **iso 16:0** | 0.067 ± 0.029 | 0.070 ± 0.033 | 0.699 |
| **iso 17:0** | 0.085 ± 0.035 | 0.088 ± 0.039 | 0.724 |
| **Sum of iso BCFA** | 0.203 ± 0.085 | 0.218 ± 0.099 | 0.501 |
| **anteiso 15:0** | 0.060 ± 0.028 | 0.064 ± 0.033 | 0.613 |
| **anteiso 17:0** | 0.154 ± 0.065 | 0.153 ± 0.060 | 0.942 |
| **anteiso 19:0** | 0.067 ± 0.026 | 0.076 ± 0.026 | 0.071 |
| **Sum of anteiso BCFA** | 0.280 ± 0.115 | 0.293 ± 0.115 | 0.641 |
| **4,8,12-M-13:0** | 0.004 ± 0.003 | 0.005 ± 0.005 | 0.797 |
| **Sum of BCFA** | 0.486 ± 0.200 | 0.515 ± 0.214 | 0.552 |
| **Saturated VLCFAs** | 0.214 ± 0.019 | 0.264 ± 0.022 | 0.018 |
| **Sum of SFA** | 32.6 ± 11.1 | 31.8 ± 10.2 | 0.493 |
| **10:1** | traces | traces | 0.222 |
| **12:1** | 0.008 ± 0.005 | 0.015 ± 0.008 | 0.010* |
| **14:1** | 0.214 ± 0.098 | 0.259 ± 0.119 | 0.123 |
| **16:1** | 4.34 ± 1.90 | 4.20 ± 1.58 | 0.760 |
| **17:1** | 0.203 ± 0.081 | 0.162 ± 0.061 | 0.020* |
| **18:1** | 49.5 ± 16.5 | 51.9 ± 15.9 | 0.050 |
| **19:1** | 0.025 ± 0.012 | 0.020 ± 0.010 | 0.121 |
| **Monounsaturated VLCFAs** | 1.05 ± 0.069 | 0.877 ± 0.103 | 0.012* |
| **20:1** | 1.007 ± 0.365 | 0.819 ± 0.300 | 0.003* |
| **22:1** | 0.034 ± 0.016 | 0.039 ± 0.035 | 0.860 |
| **24:1** | 0.009 ± 0.006 | 0.019 ± 0.011 | 0.002* |
| **Sum of MUFA** | 55.4 ± 18.5 | 57.4 ± 17.6 | 0.059 |
| **HDA 16:2 n-6** | 0.010 ± 0.006 | 0.011 ± 0.006 | 0.531 |
| **LA 18:2 n-6** | 10.7 ± 3.96 | 9.74 ± 3.07 | 0.064 |
| **ARA 20:4 n-6** | 0.321 ± 0.149 | 0.264 ± 0.164 | 0.208 |
| **DGLA 20:3 n-6** | 0.225 ± 0.116 | 0.182 ± 0.162 | 0.044* |
| **EDA 20:2 n-6** | 0.219 ± 0.088 | 0.167 ± 0.074 | 0.013* |
| **n-6 DPA 22:5 n-6** | 0.016 ± 0.010 | 0.014 ± 0.009 | 0.592 |
| **AdA 22:4 n-6** | 0.142 ± 0.088 | 0.104 ± 0.097 | 0.221 |
| **Sum of PUFA n-6** | 11.7 ± 4.23 | 10.5 ± 3.29 | 0.026 |
| **ALA 18:3 n-3** | 0.022 ± 0.011 | 0.025 ± 0.012 | 0.375 |
| **EPA 20:5 n-3** | 0.057 ± 0.032 | 0.048 ± 0.034 | 0.234 |
| **ETA 20:4 n-3** | 0.008 ± 0.004 | 0.004 ± 0.004 | 0.001* |
| **DHA 22:6 n-3** | 0.120 ± 0.091 | 0.084 ± 0.069 | 0.240 |
| **n-3 DPA 22:5 n-3** | 0.184 ± 0.121 | 0.125 ± 0.115 | 0.161 |
| **Sum of PUFA n-3** | 0.391 ± 0.245 | 0.285 ± 0.220 | 0.115 |
| **Sum of PUFA** | 12.1 ± 4.35 | 10.8 ± 3.41 | 0.022* |

Values are mean ± SD; ND, not detected; traces indicated that diagnostic ions were present but the signal was too small to be integrated. For FA abbreviations see Table 2 in the main text.

**Supplementary Table S6**. BMI-associated effects on serum fatty acid levels estimated using ANCOVA (adjusted for group)

|  | **Slope** | **Standard error** | **t-value** | **p – value** | **p – value adjusted** |
| --- | --- | --- | --- | --- | --- |
| **10:0** | < 0.001 | < 0.001 | -2.22 | 0.029* | 0.073 |
| **12:0** | -0.001 | 0.002 | -0.47 | 0.641 | 0.730 |
| **18:0** | -0.033 | 0.030 | -1.10 | 0.273 | 0.386 |
| **Even-chained VLCFAs** | -0.014 | 0.005 | -2.76 | 0.007* | 0.029* |
| **20:0** | -0.003 | 0.002 | -1.80 | 0.075 | 0.143 |
| **22:0** | -0.004 | 0.002 | -2.45 | 0.017* | 0.049* |
| **24:0** | -0.006 | 0.002 | -3.18 | 0.002* | 0.022* |
| **26:0** | -0.001 | < 0.001 | -1.91 | 0.060 | 0.128 |
| **28:0** | < 0.001 | < 0.001 | -0.99 | 0.326 | 0.424 |
| **Other ECFA** | -0.004 | 0.001 | -3.14 | 0.002* | 0.022* |
| **Sum of ECFA** | 0.139 | 0.071 | 1.96 | 0.054 | 0.120 |
| **11:0** | < 0.001 | < 0.001 | -3.26 | 0.0028* | 0.022* |
| **13:0** | < 0.001 | < 0.001 | -1.81 | 0.074 | 0.143 |
| **Odd-chained VLCFAs** | < 0.001 | < 0.001 | -1.04 | 0.301 | 0.405 |
| **21:0** | < 0.001 | < 0.001 | -1.08 | 0.283 | 0.388 |
| **23:0** | -0.002 | 0.001 | -2.99 | 0.004* | 0.023* |
| **25:0** | < 0.001 | < 0.001 | -1.59 | 0.116 | 0.187 |
| **Other OCFA** | -0.004 | 0.002 | -2.28 | 0.025* | 0.070 |
| **Sum of OCFA** | -0.012 | 0.004 | -3.49 | 0.001* | 0.022* |
| ***iso* 15:0** | < 0.001 | < 0.001 | -1.62 | 0.109 | 0.183 |
| ***iso* 16:0** | -0.001 | < 0.001 | -2.10 | 0.039* | 0.090 |
| ***iso* 17:0** | -0.003 | 0.001 | -3.10 | 0.003* | 0.023* |
| ***iso* 22:0** | < 0.001 | < 0.001 | -1.94 | 0.056 | 0.121 |
| **Other *iso* BCFA** | < 0.001 | < 0.001 | 0.10 | 0.919 | 0.948 |
| **Sum of *iso* BCFA** | -0.004 | 0.001 | -2.95 | 0.004* | 0.023 |
| **anteiso 17:0** | -0.001 | 0.001 | -1.48 | 0.143 | 0.225 |
| **anteiso 19:0** | -0.001 | < 0.001 | -1.65 | 0.104 | 0.183 |
| **anteiso 23:0** | < 0.001 | < 0.001 | -2.16 | 0.034* | 0.081 |
| **Other *anteiso* BCFA** | -0.001 | 0.001 | -1.10 | 0.277 | 0.386 |
| **Sum of *anteiso* BCFA** | -0.003 | 0.001 | -1.86 | 0.066 | 0.137 |
| **Sum of BCFA** | -0.008 | 0.003 | -2.63 | 0.010* | 0.035* |
| **Saturated VLCFAs** | -0.017 | 0.006 | -2.82 | 0.006* | 0.029* |
| **Sum of SFA** | 0.119 | 0.073 | 1.63 | 0.108 | 0.183 |
| **19:1** | < 0.001 | < 0.001 | -0.18 | 0.859 | 0.909 |
| **Monounsaturated VLCFAs** | -0.006 | 0.004 | -1.64 | 0.104 | 0.183 |
| **20:1** | < 0.001 | 0.002 | -0.09 | 0.926 | 0.948 |
| **22:1** | -0.001 | 0.001 | -0.38 | 0.705 | 0.776 |
| **24:1** | -0.005 | 0.003 | -1.84 | 0.070 | 0.140 |
| **Other MUFA** | 0.264 | 0.102 | 2.59 | 0.012* | 0.038* |
| **Sum of MUFA** | 0.258 | 0.103 | 2.51 | 0.014* | 0.044* |
| **AdA 22:4 n-6** | 0.001 | 0.001 | 0.64 | 0.522 | 0.634 |
| **Other PUFA n-6** | -0.349 | 0.127 | -2.74 | 0.008* | 0.029* |
| **Sum of PUFA n-6** | -0.349 | 0.128 | -2.73 | 0.008* | 0.029* |
| **ETA 20:4 n-3** | < 0.001 | 0.001 | 0.20 | 0.842 | 0.903 |
| **DHA 22:6 n-3** | -0.018 | 0.018 | -1.02 | 0.311 | 0.411 |
| **n-3 DPA 22:5 n-3** | -0.004 | 0.003 | -1.32 | 0.192 | 0.296 |
| **Other PUFA n-3** | -0.006 | 0.013 | -0.43 | 0.667 | 0.748 |
| **Sum of PUFA n-3** | -0.028 | 0.032 | -0.86 | 0.392 | 0.491 |
| **Sum of PUFA** | -0.376 | 0.140 | -2.69 | 0.009* | 0.031* |

Slope values represent the estimated change in fatty acid level per one-unit increase in BMI, based on ANCOVA models adjusted for group (cancer patients and healthy controls). *p-values* reflect the significance of the BMI effect. Adjusted p-values were calculated using the Benjamini–Hochberg false discovery rate (FDR) method. *** indicate p-values < 0.05. For group names refer to Table 3 in the main manuscript.

Supplementary Table S7. Comparison of VLCFAs groups content in serum stratified for stage.

| **Cancer tissue** | **Stage I**  **(n = 21)** | **Stage II and III**  **(n = 27)** | **p-value adjusted for BMI** |
| --- | --- | --- | --- |
| **Even-chained VLCFAs ≥ 20:0** | 0.584 ± 0.03 | 0.612 ± 0.054 | 0.691 |
| **20:0** | 0.156 ± 0.008 | 0.157 ± 0.021 | 0.982 |
| **22:0** | 0.202 ± 0.011 | 0.209 ± 0.017 | 0.786 |
| **24:0** | 0.195 ± 0.013 | 0.214 ± 0.018 | 0.381 |
| **26:0** | 0.025 ± 0.003 | 0.028 ± 0.002 | 0.697 |
| **28:0** | 0.006 ± 0.001 | 0.006 ± 0.001 | 0.688 |
| **Odd-chained VLCFAs ≥ 21:0** | 0.095 ± 0.006 | 0.094 ± 0.009 | 0.894 |
| **21:0** | 0.02 ± 0.002 | 0.021 ± 0.002 | 0.858 |
| **23:0** | 0.081 ± 0.005 | 0.082 ± 0.009 | 0.959 |
| **25:0** | 0.014 ± 0.002 | 0.012 ± 0.001 | 0.246 |
| **Saturated VLCFAs** | 0.700 ± 0.037 | 0.727 ± 0.064 | 0.754 |
| **Monounsaturated VLCFAs ≥ 20:1** | 0.509 ± 0.032 | 0.462 ± 0.035 | 0.330 |
| **20:1** | 0.205 ± 0.03 | 0.171 ± 0.012 | 0.324 |
| **22:1** | 0.038 ± 0.02 | 0.023 ± 0.004 | 0.472 |
| **24:1** | 0.267 ± 0.018 | 0.268 ± 0.032 | 0.979 |

Values are mean ± SD, p-value for ANCOVA with BMI as a covariate, adjusted with Benjamini-Hochberg

Supplementary Table S8. Comparison of VLCFAs groups content in serum stratified for histopathological type

| **Cancer tissue** | **Luminal A**  **(n = 20)** | **Luminal B**  **(n = 22)** | **Luminal B HER2+**  **(n = 4)** | **p-value**  **Luminal A vs Luminal B** | **p-value**  **Luminal A vs Luminal B HER2+** | **p-value**  **Luminal B vs Luminal B HER2+** |
| --- | --- | --- | --- | --- | --- | --- |
| **Even-chained VLCFAs ≥ 20:0** | 0.552 ± 0.034 | 0.680 ± 0.055 | 0.515 ± 0.092 | 0.068 | 0.992 | 0.289 |
| **20:0** | 0.146 ± 0.010 | 0.180 ± 0.022 | 0.128 ± 0.028 | 0.260 | 0.949 | 0.413 |
| **22:0** | 0.196 ± 0.013 | 0.228 ± 0.018 | 0.185 ± 0.031 | 0.218 | 0.993 | 0.488 |
| **24:0** | 0.183 ± 0.013 | 0.234 ± 0.018 | 0.175 ± 0.033 | 0.029* | 0.997 | 0.275 |
| **26:0** | 0.023 ± 0.003 | 0.030 ± 0.002 | 0.025 ± 0.010 | 0.152 | 0.888 | 0.766 |
| **28:0** | 0.005 ± 0.002 | 0.008 ± 0.001 | 0.003 ± 0.002 | 0.245 | 0.829 | 0.266 |
| **Odd-chained VLCFAs ≥ 21:0** | 0.109 ± 0.008 | 0.128 ± 0.011 | 0.106 ± 0.020 | 0.226 | 0.995 | 0.626 |
| **21:0** | 0.019 ± 0.002 | 0.023 ± 0.002 | 0.023 ± 0.002 | 0.319 | 0.678 | 0.999 |
| **23:0** | 0.077 ± 0.006 | 0.091 ± 0.009 | 0.075 ± 0.018 | 0.284 | 0.991 | 0.698 |
| **25:0** | 0.013 ± 0.002 | 0.014 ± 0.002 | 0.008 ± 0.002 | 0.767 | 0.554 | 0.317 |
| **Saturated VLCFAs** | 0.666 ± 0.041 | 0.808 ± 0.064 | 0.621 ± 0.112 | 0.078 | 0.996 | 0.325 |
| **Monounsaturated VLCFAs ≥ 20:1** | 0.489 ± 0.036 | 0.506 ± 0.039 | 0.393 ± 0.042 | 0.915 | 0.547 | 0.403 |
| **20:1** | 0.202 ± 0.033 | 0.168 ± 0.012 | 0.195 ± 0.042 | 0.614 | 0.992 | 0.892 |
| **22:1** | 0.041 ± 0.023 | 0.023 ± 0.005 | 0.018 ± 0.003 | 0.748 | 0.834 | 0.991 |
| **24:1** | 0.247 ± 0.017 | 0.316 ± 0.035 | 0.180 ± 0.049 | 0.148 | 0.598 | 0.095 |

Values are mean ± SD, p-value for ANCOVA with BMI as a covariate, followed by post hoc pairwise comparisons between subtypes using estimated marginal means, adjusted using Tukey’s method. * denotes p-value < 0.05.

**
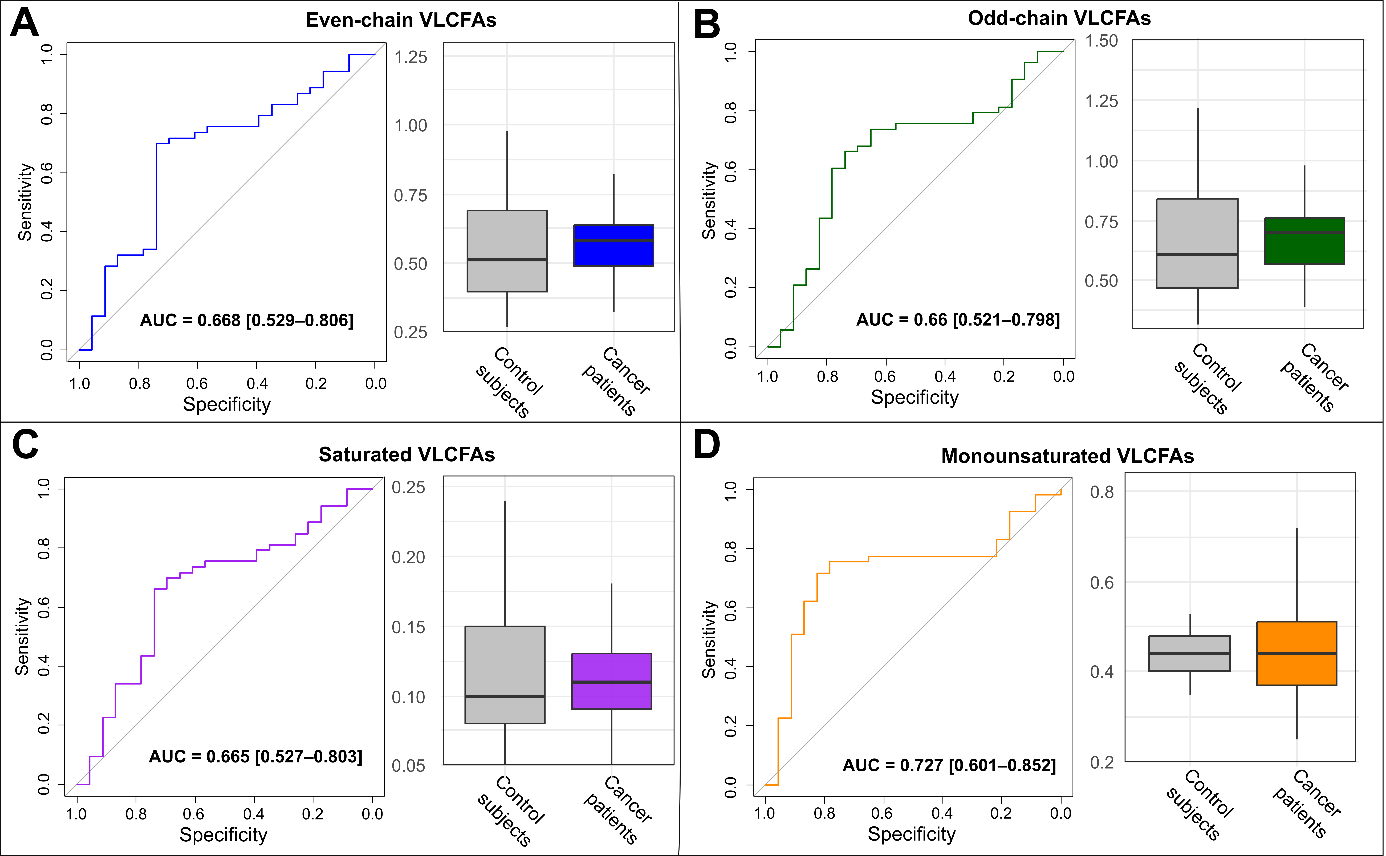
**

Supplementary Figure S5 ROC curves and corresponding boxplots of serum content of different FA groups with very long chains

Plotted is sum of content of A: VLCFAs with even, saturated chains, p = 0.776; B: VLCFA with odd, saturated chains, p = 0.928; C: saturated VLCFAs, p = 0.840; D: monounsaturated VLCFAs, p = 0.922. Values are adjusted for BMI. AUC, area under curve given with 95% confidence interval in parenthesis.
